# How frail is frail? A systematic scoping review and synthesis of high impact studies

**DOI:** 10.1186/s12877-021-02671-3

**Published:** 2021-12-18

**Authors:** E. H. Gordon, N. Reid, I. S. Khetani, R. E. Hubbard

**Affiliations:** 1 Centre for Health Services Research, The University of Queensland, Building 33, Princess Alexandra Hospital, 199 Ipswich Road, Woolloongabba, QLD 4102 Australia; 2 Princess Alexandra Hospital, Metro South Hospital and Health Service, Woolloongabba, Queensland Australia

**Keywords:** Frailty, Frailty index, Deficit accumulation

## Abstract

**Aims:**

While the frailty index (FI) is a continuous variable, an FI score of 0.25 has construct and predictive validity to categorise community-dwelling older adults as frail or non-frail. Our study aimed to explore which FI categories (FI scores and labels) were being used in high impact studies of adults across different care settings and why these categories were being chosen by study authors.

**Methods:**

For this systematic scoping review, Medline, Cochrane and EMBASE databases were searched for studies that measured and categorised an FI. Of 1314 articles screened, 303 met the eligibility criteria (community: *N* = 205; residential aged care: *N* = 24; acute care: *N* = 74). For each setting, the 10 studies with the highest field-weighted citation impact (FWCI) were identified and data, including FI scores and labels and justification provided, were extracted and analysed.

**Results:**

FI scores used to distinguish frail and non-frail participants varied from 0.12 to 0.45 with 0.21 and 0.25 used most frequently. Additional categories such as mildly, moderately and severely frail were defined inconsistently. The rationale for selecting particular FI scores and labels were reported in most studies, but were not always relevant.

**Conclusions:**

High impact studies vary in the way they categorise the FI and while there is some evidence in the community-dweller literature, FI categories have not been well validated in acute and residential aged care. For the time being, in those settings, the FI should be reported as a continuous variable wherever possible. It is important to continue working towards defining frailty categories as variability in FI categorisation impacts the ability to synthesise results and to translate findings into clinical practice.

**Supplementary Information:**

The online version contains supplementary material available at 10.1186/s12877-021-02671-3.

## Introduction

Over the last decade, there has been exponential growth in the number of ‘FI studies’ published in peer-reviewed journals. The frailty index (FI) represents the accumulated deficit model of frailty [1] and is a continuous variable (ranging from zero to a theoretical maximum of one) derived from a list of potential health deficits [2]. Increasingly, FI scores are being used to assign individuals to frailty categories.

In their 2007 study, Rockwood and colleagues [3] found that an FI = 0.25 was the ‘crossing point’ of robust and frail groups (as measured by the phenotypic model of frailty) and predicted death and institutionalisation. These results were consistent with findings of an earlier study by this group. In 2005, Rockwood et al. [4] showed that the FI and Clinical Frailty Scale (CFS; a scale of increasing functional dependence) were highly correlated and independently predicted adverse outcomes, and that an FI = 0.25 lay between CFS category 4 (‘apparently vulnerable’, mean FI = 0.22) and CFS category 5 (‘mildly frail’, mean FI = 0.27). Together, Rockwood et al.’s studies demonstrated that an FI = 0.25 had construct and predictive validity to categorise community-dwelling older adults as frail or non-frail.

Nevertheless, a variety of FI categories have emerged in the literature. Our study had two key aims: firstly, to explore which FI categories (FI scores and labels) were being used in high impact studies of adults in the community, residential aged care and acute care; and secondly, why these categories were being chosen by study authors.

## Methods

### Protocol and registration

This systematic scoping review was conducted according to the Preferred Reporting Items for Systematic Reviews and Meta-Analyses extension for Scoping Reviews (PRISMA-ScR) criteria [5]. The protocol was registered with the Open Science Framework Registry.

### Search strategy

A search of Medline, Cochrane and EMBASE databases was conducted in May 2020 and again in March 2021. Search terms included ‘frailty index’, ‘acute care hospital’, ‘community’ and ‘residential care’. The full search strategy is included in the Appendix.

### Eligibility criteria

Studies were eligible for inclusion if they used an FI that met the criteria as set out by Searle and colleagues [2] and the FI was categorised in some way (i.e., an FI score(s) delineated labelled sub-categories). Included studies could be of any design, but were to be conducted in a human adult population in one of three settings: community, acute care or residential aged care. Studies were excluded if they were not an original study (e.g., a protocol or review paper), if only the abstract was available or if there were not written in English.

### Study selection

After removing duplicates, one reviewer (IK) independently screened the record titles and abstracts. Two reviewers (IK, NR) independently screened the full-text articles and disagreements were resolved by consensus and discussion with a third reviewer as required. Eligible studies were separated into the three settings of interest. A field-weighted citation impact (FWCI) score was calculated for each study. Sourced from SciVal, the FWCI compares the number of citations a publication receives to the average number of citations received by other *similar publications* in the Scopus database [6]. Similar publications are those that have the same publication year, publication type and discipline. Consequently, newer publications are not disadvantaged using this methodology. The ten studies with the highest FWCIs (i.e., the 10 ‘highest impact’ studies) from each setting underwent data extraction.

### Data extraction and analysis

Three reviewers (IK, NR, EG) performed data extraction and any disagreements were resolved by consensus. Extracted study data included country, publication date, study design and sample size. FI data included mean, FI scores and labels and justification provided by the study author(s) for these FI categories.

## Results

### Study characteristics

**Fig. 1:**
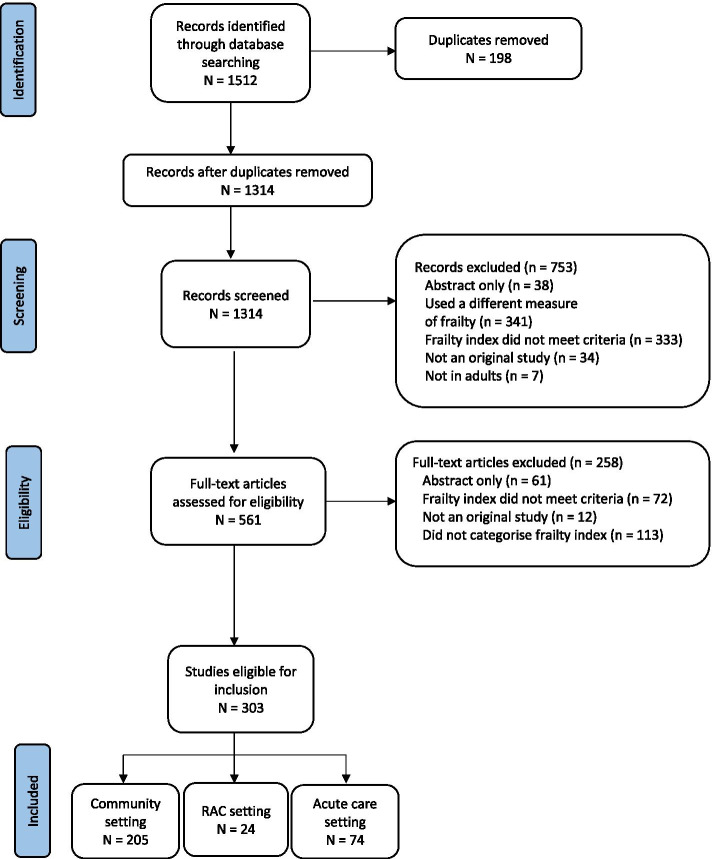
PRISMA diagram of study selection

The search strategy yielded 1512 studies and 303 were eligible for inclusion (Fig. 1). Of the 30 highest impact studies (i.e., 10 highest impact studies from each setting), 29 were published in the last decade (Table 1). Twenty-one studies were cohort design and seven were cross-sectional. The majority were conducted in North America. Study sample size ranged from 50 to 931,541. The mean FI of the populations described in the studies ranged from 0.07 to 0.42.

**Table 1:** Results: Community, acute care and residential aged care settings

	Author, Year, Country	FCWI	Study Design	N	Mean Age in years (SD)	% Female	Mean FI (SD)	FI Categories: scores and labels	Rationale & Comments
**Community**	Clegg et al., 2016 UK [7]	19.42	Cohort	931,541	75.0 (7.2)	55.0	Development and internal validation cohort = 0.14 (0.09) External validation cohort = 0.15 (0.10)	< 0.12 (fit) > 0.12–0.24 (mild frailty) > 0.24–0.36 (moderate frailty) > 0.36 (severe frailty)	Study authors derived FI quartiles using 99th centile as upper limit.
	Wallace et al., 2019 USA [8]	17.94	Cross-sectional	456	89.7 (6.1)	69.0	0.42 (0.18)	≥ 0.41 (median) (high frailty) 0.24–0.41 (low frailty) 0.42 (intermediate frailty) 0.43–0.60 (high frailty)	Study authors derived FI categories utilising the median and then using the mean + − 1 SD.
	Rockwood et al., 2011 Canada [9]	17.78	Cohort	14,127	44.3 (18.3)	54.2	0.07 (0.08)	≤ 0.03 (relatively fit) 0.03 < FI ≤ 0.10 (less fit) 0.10 < FI ≤ 0.21 (least fit) > 0.21 (frail) ≥ 0.45 (most frail)	Referenced Rockwood et al.’s study [4], which demonstrated construct and predictive validity of Clinical Frailty Scale (CFS) categories in study of community-dwellers. CFS categories ≥4 (‘apparently vulnerable’- ‘severely frail’) corresponded with a mean FI > 0.21.
	Theou et al., 2013 11 European countries [10]	11.00	Cohort	27,527	65.3 (10.5)	54.8	Not reported	≥ 0.25 (frail)	Referenced Rockwood et al.’s study [3], which demonstrated the construct and predictive validity of FI > 0.25 in community-dwellers.
	Blodgett et al., 2015 Canada [11]	8.52	Cross-sectional	4096	63.4 (10.3)	53.3	0.20 men 0.17 women	≤ 0.10 (non-frail) 0.10 < FI ≤ 0.21 (vulnerable) 0.21 < FI ≤ 0.45 (frail) > 0.45 (most frail)	Referenced Hoover et al.’s study [12], which demonstrated the predictive validity of FI > 0.21 cut-off as well as four frailty categories (as listed here).
	Thompson et al., 2018 Australia [13]	7.23	Cohort	909	74.4 (6.2)	55.0	0.23 (0.15)	≤ 0.21 (non-frail and prefrail) > 0.21 (frail and most frail)	Referenced Hoover et al. [12]
	Ntanasi et al., 2018 Greece [14]	6.76	Cross-sectional	1740	73.4 (5.4)	59.0	Not reported	> 0.25 (frail)	Referenced Rockwood et al. [3]
	Song et al., 2010 Canada [15]	6.51	Cohort	2740	74.0 (6.6)	60.8	Not reported	≤ 0.08 (non-frail) 0.09–0.24 (prefrail) ≥ 0.25 (frail)	Referenced Rockwood et al. [3]
	Ravindrarajah et la., 2017 UK [16]	6.00	Cohort	144,403	85.1 (4.9) – 88.0 (5.4)	50–68	Not reported	< 0.12 (fit) > 0.12–0.24 (mild frailty) > 0.24–0 36 (moderate frailty) > 0.36 (severe frailty)	Referenced Clegg et al.’s study [7], which demonstrated the predictive validity of these eFI categories in UK community-dwellers.
	Lansbury et al., 2017 UK [17]	5.22	Cross-sectional	589	82.7	58.1	0.23 (0.12)	< 0.12 (fit) > 0.12–0.24 (mild frailty) > 0.24–0.36 (moderate frailty) > 0.36 (severe frailty)	Referenced Clegg et al. [7]
**Acute Care**	Joseph et al., 2014 USA [18]	15.46	Cohort	250	77.9 (8.1)	30.8	0.21 (0.10)	< 0.25 (non-frail) ≥ 0.25 (frail)	Referenced Searle et al.’s study [2], which did not report FI categories.
	Chong et al., 2018 Singapore [19]	5.49	Cohort	210	89.4 (4.6)	69.5	Not reported	≥ 0.25 (frail)	Nil
	Joseph et al., 2016 USA [20]	5.03	Cohort	220	75.5 (7.7)	44.0	0.28 (0.13)	< 0.25 (non-frail) ≥ 0.25 (frail)	Referenced study by co-authors [18] and a conference abstract.
	Poudel et al., 2016 Australia [21]	4.93	Cohort	1418	81 (6.8)	55.0	0.32 (0.15)	< 0.25 (low) 0.26–0.39 (medium) ≥ 0.4 (high)	Referenced Rockwood et al. [3, 4] Also referenced Singh et al.’s study [22], which utilised similar categories and referenced Rockwood et al. [3, 4]
	Andrew et al., 2017 Canada [23]	4.87	Case control	884	78.8 (7.9) – 80.6 (9.0)	55.0–56.9	Cases = 0.2 (0.11) Controls = 0.22 (0.13)	< 0.10 (non-frail) > 0.10–0.21 (prefrail) > 0.21–0.45 (frail)	Referenced Hoover et al. [12]
	Dent et al., 2014 Australia [24]	4.22	Cohort	172	Not reported	72.0	Not reported	< 0.2 (robust) 0.2–0.45 (prefrail) > 0.45 (frail)	Referenced Rockwood et al. [4]
	Mueller et al., 2016 USA [25]	4.16	Cohort	102	61.9 (15.8)	39.2	0.23 (0.12)	< 0.25 (non-frail) ≥ 0.25 (frail)	Referenced Joseph et al. [18]
	Zeng et al., 2015 China [26]	2.92	Cohort	155	82.7 (7.1)	12.9	Not reported	< 0.22 (least frail) > 0.46 (least fit)	Authors determined FI scores below which all participants survived and above which all participants died.
	Hao et al., 2019 China [27]	2.86	Cohort	271	81.1 (6.6)	20.3	0.26 (0.16)	> 0.25 (frail)	Referenced Rockwood et al. [3] Also referenced several other studies that utilised the same categories and referenced Rockwood et al. [3, 4].
	Arjunan et al., 2019 Australia [28]	2.83	Cohort	258	79.0 (8.0)	54.0	0.42 (0.13)	≤ 0.40 (less frail) > 0.40 (more frail)	Authors determined the FI cut point for optimal sensitivity and specificity for four adverse outcomes.
**Residential Aged Care**	Theou et al., 2018 Spain [29]	4.00	RCT	50	75.3 (7.3)	70.0	0.23 (0.1)	< 0.20 (non-frail) 0.20–0.30 (vulnerable/mildly frail) > 0.30 (moderately/severely frail)	Study authors categorised the FI in 0.1 groups then combined groups due to the small number of participants. They referenced two studies [30, 31], which both categorised the FI into 0.1 increments to facilitate regression analyses.
	Shaw et al., 2019 Canada [32]	3.84	Cohort	116	84.2 (0.9)	56.0	0.36 (0.01)	< 0.27 (non-frail) ≥ 0.27 (frail)	Study authors demonstrated a bimodal distribution of the continuous FI with ‘crossing points’ at an FI = 0.27.
	Theou et al., 2018 Australia [33]	3.26	Cohort	383	Median 88.0 IQR 4.0	77.6	0.33 (0.24–0.46)	≤ 0.10 (non-frail) 0.10–0.21 (vulnerable) 0.21–0.44 (mild/moderate frailty) ≥ 0.45 (most frail)	Referenced study by co-authors [34], which utilised the same categories and referenced Hoover et al. [12]
	Maclagan et al., 2017 Canada [35]	2.33	Cohort	41,351	Not reported	64.7	Not reported	< 0.20 (robust / non-frail) 0.20–0.30 (pre-frail) > 0.30 (frail)	Referenced study by co-authors [36], which utilised the same FI categories, referencing Searle et al. [2], co-authors Hogan et al. [37] (see below) and Kulminski et al. [38] Kulminski et al.’s study [38] demonstrated the predictive validity of similar FI categories in community-dwellers.
	Hogan et al., 2012 Canada [37]	2.03	Cohort	1066	84.9 (7.3)	76.7	Not reported	< 0.20 (robust / non-frail) ≥ 0.20 ≤ 0.30 (prefrail) > 0.30 (frail)	Referenced Searle et al. [2] and Kulminski et al. [38]
	Buckinx et al., 2017 Belgium [39]	1.24	Cohort	662	83.2 (9.0)	72.5	Not reported	< 0.25 (robust) ≥ 0.25 (frail)	Referenced a review article [40] and Mitnitski et al.’s study [41], which based ‘FI-Biomarker’ categories on maximum separation of mortality curves in community-dwellers.
	Ambagtsheer et al., 2020 Australia [42]	1.23	Cross-sectional	592	Median 88.0 IQR 9.0	66.6	0.20 (0.08)	≤ 0.10 (non-frail) > 0.10 ≤ 0.21 (pre-frail) > 0.21 (frail)	Referenced Hoover et al. [12]
	Ambagtsheer et al., 2020 Australia [43]	1.03	Cross-sectional	592	Median 88.0 IQR 9.0	66.6	Not reported	≤ 0.21 (non-frail) > 0.21 (frail)	Referenced Hoover et al. [12]
	Ge et al., 2019 China [44]	0.72	Cross-sectional	302	82.7 (8.5)	71.2	0.27 (0.11)	< 0.21 (non-frail) 0.22–0.44 (frail) ≥ 0.45 (frailest)	Referenced Hoover et al. [12]
	Stock et al., 2017 Canada [45]	0.54	Cohort	1066	84.4 (7.3)	76.7	Not reported	< 0.20 (non-frail) 0.20–0.30 (prefrail) > 0.30 (frail)	Referenced study by co-authors [37] (see above).

Note: *FWCI* field-weighted citation impact as at 31st March 2021

### FI categories

In studies of community-dwelling adults, an FI = 0.25 delineated frail and non-frail individuals in three studies [10, 14, 15], all of which referenced Rockwood and colleagues’ 2007 study [3]. An FI = 0.21 was used in three studies [9, 11, 13]. One referenced Rockwood et al.’s CFS validation study [4] and the other two referenced Hoover and colleagues’ study [12], which demonstrated the predictive validity of this FI cut-off in older community-dwellers. In a large cohort study using the electronic FI (eFI), Clegg et al. [7] used quartiles to define fit (FI < 0.12) versus frail (FI > 0.12) categories. Subsequently, two high impact UK studies adopted these eFI categories for their analyses [16, 17].

In the acute care setting, an FI = 0.25 was the most common score used to determine frailty [18–20, 25, 27]. One study referenced Rockwood and colleagues’ community-dweller study [4]. The other studies either provided no justification, referenced studies that did not use FI categories or referenced other papers written by the same authors. Incident adverse outcomes were used to delineate frailty severity (i.e., less or more frail; least frail and least fit) in two studies [26, 28].

In studies of adults residing in residential aged care, there was even greater variability. One study defined frailty as an FI ≥ 0.25 [39] and referenced studies that did not evaluate the validity of this cut-off. Four studies utilised an FI = 0.21 to define frailty [42–44] and all referenced (directly or indirectly) the community-dweller study by Hoover et al. [12]. Three studies defined frail as an FI > 0.30 [35, 37, 45]. Two referenced other papers written by the same authors and one referenced a study that demonstrated the predictive validity of similar FI categories in community-dwellers [38].

Across the settings, additional categories such as robust, pre-frail, mildly, moderately and severely frail were defined inconsistently. Methods included examining data spread (such as FI quartiles) [7, 8, 16, 17, 29, 32] and sensitivity/specificity analyses (in relation to adverse outcomes) [26, 28]. Three studies [11, 33, 44], two of which were conducted in residential aged care, adopted the categories that Hoover et al. [12] validated.

## Discussion

This scoping review demonstrated variability in FI categorisation in high impact studies of community-dwellers, acute care patients and adults living in a residential aged care. An FI = 0.25 was the most commonly used score to determine frailty, although this was used in less than half of all studies. Greatest variability was seen in residential aged care studies. The rationale for using particular FI categories was reported in most studies, but was not always relevant.

Fourteen studies referenced Rockwood et al. [3, 4] and Hoover et al. [12] as justification for a variety of FI cut-offs and labels. Researchers used the mean FI values reported in Rockwood and colleague’s CFS study [4] to define FI categories, but not all in the same way. While some categories (e.g., frail = FI > 0.21 versus FI > 0.25) were similar, others (e.g., frail = FI > 0.45 versus most frail = FI ≥ 0.45) probably captured different groups of adults. In their 2013 study, Hoover and colleagues [12] tested the predictive validity of published cut-offs (including FI > 0.21 [4], > 0.25 [3] and > 0.35 [38]) in an older community-dwelling population. Using stratum-specific likelihood ratios for hospital-related outcomes, they identified four frailty categories (non-frail = FI < 0.1, pre-frail = 0.1 < FI ≤ 0.21, frail = FI > 0.21 and most frail = FI ≥ 0.45). These categories align with Rockwood et al.’s study [4], where the mean FIs of very fit (CFS 1) and severely frail (CFS 7) adults were 0.09 and 0.43, respectively.

Some FI categories validated in community-dwelling populations have been used in studies of adults in acute and residential aged care. It is debatable whether FI categories should vary by setting. Certainly, in these care settings, a greater proportion of adults are frail and, as a result, dichotomizing the FI into frail and non-frail is suboptimal. For example, in their recent cross-sectional study of Australian aged care residents, Ambagtsheer and colleagues [42] found that using an FI score of 0.21 to delineate frail and non-frail residents yielded a frailty prevalence rate of 43.6%. Thus, the heterogeneity of almost half of the residents’ health statuses would not be captured using this categorisation.

Frailty prevalence rates are also high in the acute setting. For example, Joseph and colleagues [18] found that 44% of geriatric trauma patients were frail (FI > 0.25). In a previous study by our group [46], the negative predictive value for an FI > 0.40 was high (84–98%) for all adverse outcomes, including individual geriatric syndromes, in older inpatients. This study was not included in this scoping review as the authors did not use this FI value to define FI categories (such as FI > 0.4 = more frail or FI < 0.40 = less frail). Nevertheless, two studies included in this review yielded similar results: an FI > 0.46 and an FI > 0.40 predicted adverse outcomes in elderly patients in intensive care and rehabilitation, respectively [26, 28]. These data indicate that an FI ≥ 0.40 is a valid cut-off for severe frailty in the acute care setting. Overall, further data are required to validate mild, moderate and severe categories and to determine whether these categories are applicable across settings.

The major limitation of this scoping review is that data were extracted from 11% of eligible studies. The decision to extract data from the studies with the highest FWCIs was primarily pragmatic. This study not only aimed to describe *which* FI categories were being used in the literature but also aimed to examine *why* these categories were being chosen. It was not feasible to extract and present data with this degree of granularity from over 300 studies. Studies with the highest FWCIs are most likely to influence and to have influenced adoption of FI categories in clinical practice and research. Therefore, extracting and synthesising data from these studies generates meaningful results relevant to both spheres. Overall, this methodology yielded highly heterogeneous results and it is unlikely that extracting data from more studies would have resulted in consensus regarding FI categorisation. An additional limitation of this systematic scoping review is that only one reviewer screened titles and abstracts.

In summary, this scoping review demonstrated that high impact studies vary in the way they categorise the FI and while there is some evidence in the community-dweller literature, FI categories have not been well validated in acute and residential aged care. For the time being, the FI should be reported as a continuous variable wherever possible. It is important to continue working towards defining frailty categories - it may be desirable for researchers to recruit only mildly frail community-dwellers for an intervention study or it may be preferable for hospital-based clinicians to provide severely frail patients with an alternative model of care to mildly frail patients. Variable, unvalidated FI categorisation impacts the ability to synthesise results and to translate findings into clinical practice.

## Supplementary Information

**Additional file 1.**

## Data Availability

Data sharing is not applicable to this study as no datasets were generated for analysed during the current study.

## Abbreviations

FI: frailty index
CFS: Clinical Frailty Scale
FWCI: field-weighted citation impact
RAC: residential aged care
SD: standard deviation
eFI: electronic frailty index
