# Supplementary material for: How frail is frail? A systematic scoping review and synthesis of high impact studies
Source: BMC Geriatr. 2021 Dec 18;21:719. doi: 10.1186/s12877-021-02671-3 (PMC8684089; doi:10.1186/s12877-021-02671-3)
Supplement: Supplementary file 1 — Additional file 1. [file 12877_2021_2671_MOESM1_ESM.docx]

**APPENDIX**

**Search Strategy**

The following search strategy was implemented in all databases using keywords in title and abstract sections:
(1) frailty index OR Rockwood frailty score*;*

(2) health deficit or accumulated deficit NOT paediatric / ADHD / attention deficit / child*;*

(3) acute care hospital OR emergency department OR emergency room OR community OR nursing home OR long term care OR residential care OR aged care;

(4) (1) AND (3);

(5) (2) AND (3);

(6) (4) OR (5)
